# Characterization of a Graphene Oxide-Reinforced Whey Hydrogel as an Eco-Friendly Absorbent for Food Packaging

**DOI:** 10.3390/gels9040298

**Published:** 2023-04-03

**Authors:** Pompilia Mioara Purcea Lopes, Dumitrita Moldovan, Radu Fechete, Liviu Mare, Lucian Barbu-Tudoran, Niculina Sechel, Violeta Popescu

**Affiliations:** 1 Physics and Chemistry Department, Technical University of Cluj-Napoca, 28 Memorandumului Str., 400114 Cluj-Napoca, Romania; dumitrita.moldovan@phys.utcluj.ro (D.M.); rfechete@phys.utcluj.ro (R.F.); mareliviumarius@gmail.com (L.M.); 2 Electron Microscopy Center, Faculty of Biology and Geology, Babes-Bolyai University of Cluj-Napoca, 1 M. Kogalniceanu Street, 400347 Cluj-Napoca, Romania; lucian.barbu@ubbcluj.ro; 3 Materials Science and Engineering Department, Technical University of Cluj-Napoca, 103-105 Muncii Avenue, 400641 Cluj-Napoca, Romania; niculina.sechel@stm.utcluj.ro

**Keywords:** whey, gelatin, hydrogel, graphene oxide, cross-linking, food packaging

## Abstract

This study presents a structural analysis of a whey and gelatin-based hydrogel reinforced with graphene oxide (GO) by ultraviolet and visible (UV-VIS) spectroscopy, Fourier transform infrared spectroscopy (FT-IR), and X-ray diffraction (XRD). The results revealed barrier properties in the UV range for the reference sample (containing no graphene oxide) and the samples with minimal GO content of $0.66\times 10^{-3}$% and $3.33\times 10^{-3}$%, respectively, in the UV-VIS and near-IR range; for the samples with higher GO content, this was $6.67\times 10^{-3}$% and $33.33\times 10^{-3}$% as an effect of the introduction of GO into the hydrogel composite. The changes in the position of diffraction angles 2*θ* from the X-ray diffraction patterns of GO-reinforced hydrogels indicated a decrease in the distances between the turns of the protein helix structure due to the GO cross-linking effect. Transmission electron spectroscopy (TEM) was used for GO, whilst scanning electron microscopy (SEM) was used for the composite characterization. A novel technique for investigating the swelling rate was presented by performing electrical conductivity measurements, the results of which led to the identification of a potential hydrogel with sensor properties.

## 1. Introduction

Hydrogels are three-dimensional structures of polymeric materials that have the ability to absorb large amounts of liquid when submerged in an aqueous environment. This property gives them the potential to simulate living tissue and to be biodegradable and biocompatible, making them attractive in a wide range of fields such as tissue engineering [1], controlled delivery and transport systems [2], industrial wastewater treatment [3], food technology [4,5], and agriculture [6]. The strongly hydrophilic functional groups (hydroxyl, carboxyl, carbonyl, etc.) on the polymer chains allow a large amount of liquid to penetrate between the polymer chains to a level at which they can break, causing the hydrogel to dissolve. Therefore, many studies have investigated improvements in the entrapment capacity of the absorbed liquid by cross-linking the hydrogels with various compounds such as graphene oxide (GO) [7,8,9,10,11], copper compounds [12,13,14,15], alkaline salts [16,17], nanoparticles [18,19,20,21], and organic acids [22]. In addition, these materials impart specific properties that allow hydrogel formulations to meet desired application requirements such as antimicrobial properties, which are mandatory for food packaging [7,14,23], UV-VIS barriers for light-sensitive food [23,24], or water and gas barriers [23] to extend the shelf-life of food.

In the last few years, a major concern regarding the impact of packaging materials on the environment has led to the need of replacing materials based on petrochemical products such as polyethylene (PE), polypropylene (PP), polystyrene (PS), or polyethylene terephthalate (PET) with biodegradable materials based on renewable sources [23,24]. Food packaging materials have a very short life and because they are contaminated with organic biodegradable waste, they cannot be subjected to selective collection for mechanical, chemical, or energy recycling, thus ending their life in wastefields. This is the reason for the extensive research on obtaining new biodegradable and bioassimilable materials that are safe for applications in direct contact with food products.

Gelatin is one of the most studied compounds for food packaging, mostly due its ability to form films that protect, maintain, and/or extend the shelf-life of food products [25]. As an outer layer, it isolates the food from the action of light and oxygen. Gelatin can be used to obtain edible films [26,27,28,29] for meat products [30,31] or post-harvest fruit and vegetable [32,33,34,35] preservation. Mechanical properties such as fragility, rigidity, and brittleness are common in gelatin films, which often require the addition of other compounds to improve their performance such as chitosan [36,37,38,39], sodium alginate [40], grapefruit-seed extract and TiO_2_ [41], starch [42], and plant extracts [43,44]. Whey is a water-soluble compound, with the highest proportion in the composition of milk, in which valuable proteins are dissolved, conferring it with an appreciable nutritional value. As whey is produced in considerable amounts during milk processing and often treated as waste, it can serve as an inexpensive and accessible source of raw material for protein polymers. The globular proteins present in whey—namely, α-Lactalbumin, β-Lactoglobulin, and serum albumins [45]—are the main components involved in the whey gelation process, both through hydrophilic functional groups (-OH, -CONH, -CONH_2_, -COOH, and -SO_3_H) and ionic groups. Whey imparts poor mechanical qualities to hydrogels, but, by tuning with different reinforcing materials, it is a valuable precursor for hydrogels [7,14,15,46,47,48].

Gelatin- and whey protein-based materials have been rarely approached in research regarding the production of packaging materials; however, obtaining and the characterization of edible films have been described in the literature [29,49,50]. A composite with antibacterial properties based on whey protein that combined gelatin, nanoclay, orange-peel extract, and tripolyphosphate was studied by Shams [51]. They concluded that the incorporation of orange-peel extract in the film led to a reduction in transparency and an increase in the flexibility of the films.

Graphene oxide (GO) is a dark-brown powder with a two-dimensional, layered structure and a high degree of hygroscopicity. Due to the functional groups on its surface and the small size of its particles, the literature presents it as a successful binding material for different substances such as drugs [52], pollutants [53], metal nanoparticles [54], and enzymes [55] in hydrogel formulations with a wide range of polymers. Graphene is a fine solid with a high mechanical strength, which makes it ideal as a reinforcing or cross-linking material for natural polymers, known for their poor mechanical properties. Natural polymers are preferred to those from petroleum sources for their biodegradability and biocompatibility with natural food products and the human body. A widely consumed food product is milk, whose composition includes two groups of proteins: insoluble (casein) and soluble (whey and serum proteins) [36]. In addition to their nutritional qualities, whey proteins have a chemical structure that allows them to form three-dimensional gels whose functionalities can be modulated by the interaction of functional groups with other materials of protein, carbohydrate, lipid, or mineral origin, or other synthetic polymers [5]. Gelatin is a complex of proteins and polypeptides, resulting from the thermal denaturation of collagen obtained from by-products of meat and fish processing [56]. Similar to whey, gelatin proteins are characterized by a wide range of qualities: their nutritional value, high hydration and gelation capacity, biocompatibility, and biodegradability. The proteins of both precursors exhibit mechanical properties requiring reinforcement with materials such as graphene oxide [11,57], nanoparticles [14,58,59,60], nanofibers [60], hydroxyapatite [61], and montmorillonite [62].

One of the most used plasticizers in hydrogel formulas is glycerin, due to its ability to interrupt the polymer–polymer bonds through the hydrogen bonds whilst maintaining a suitable distance between the polymer chains for the absorption of fluids from the swelling medium [36]. The results of a previous study by the authors showed that this plasticizer improved the swelling capacity of a whey–gelatin composite hydrogel filled with copper particles in small amounts (9.09%) [63], as also demonstrated in other studies [36]. 

Graphene derivatives are widely studied in numerous research domains as colloids [64], reinforcers [65], electrodes [66], transport systems [52], and gas barriers [65] in hydrogel systems on their own or blended with various natural or synthetic polymers. However, little research has presented these carbon-based materials in hydrogel systems according to a review published in 2020, which reported only 9 articles characterizing graphene derivatives in food packaging systems out of 234 results displayed on the Web of Science on this topic [11]. Combining the physical, chemical, and antimicrobial qualities of the above-mentioned materials in an eco-friendly formula becomes a subject that opens up unlimited research opportunities with applications in food packaging. The most frequently used biopolymer matrixes in graphene oxide-reinforced hydrogel formulations are polylactic acid, cellulose, and chitosan, as indicated by other authors in their reviews and articles [11,67], followed by sodium alginate and starch [68]. 

One of the possibilities to reduce food waste is to extend the shelf-life of food, especially food with a high moisture content. Slicing or chopping food into smaller pieces can encourage responsible consumer behavior, but it can also lead to the release of cellular fluids that provide a nutritive medium for the growth of spoilage microorganisms. To mitigate this risk, packaging systems can be used that reduce the amount of fluids released by the food, thus limiting the growth of microbes [69].

In our previous article [7], we proposed an innovative hydrogel formulation based on whey and gelatin reinforced with graphene oxide in different concentrations as a potential absorbent material for packaging food with a high moisture content. The swelling results showed a decrease in the swelling degree in relation to the increase in GO concentration as an effect of cross-linking. This conclusion was supported by the increased stiffness of the hydrogels with the maximum GO concentration as well as shifts to lower values of T_2_ distributions in the ^1^H NMR diagrams of the hydrogels with a high GO content, corresponding with smaller pores between the polymer chains. Different GO concentrations led to different properties of whey- and gelatin-based hydrogels, as follows: (i) a maximum swelling degree was obtained at $0.66\times 10^{-3}$%, the minimal concentration of GO (sample I); (ii) at $3.33\times 10^{-3}$% GO concentration, sample II showed an antibacterial activity against *Escherichia coli* and *Salmonella enteriditis* strains; and (iii) samples III and IV at $6.67\times 10^{-3}$% and $33.33\times 10^{-3}$% GO concentrations showed an antibacterial activity against *Lysteria monocytogenes* in complement to sample II. 

This article presents a study that characterizes the properties of a nanocomposite hydrogel based on whey and gelatin reinforced with graphene oxide in different concentrations by means of spectroscopic analyses: optical absorption in the UV-VIS wavelength range, Fourier transform infrared (FT-IR), X-ray diffraction (XRD), and scanning electron microscopy (SEM). The distribution, size, and morphology of graphene oxide particles are characterized by a laser analysis and transmission electron microscopy (TEM). The swelling kinetics investigated in the authors’ previous work [7] by Fick and Schott mathematical equations and ^1^H NMR relaxometry, as well as the inhibition activity against the growth of meat-contamination bacterial strains, complement the study in this paper on whey–gelatin-based hydrogels cross-linked with graphene oxide.

## 2. Results and Discussion

### 2.1. UV-VIS Spectroscopy

The UV (300–400 nm), visible (400–700 nm), and near-infrared (700–900 nm) transmittance spectra of the nanocomposite hydrogel samples were measured in order to investigate the influence of different GO concentrations on their light transmittance properties.

The optical properties (transmission, absorption, or reflection) of substances or materials such as particles, solutions, films, colloidal dispersions, or composites depend on the concentration, morphology, and size. The color of materials is influenced by the size of particles [70,71,72] and the swelling degree.

The optical properties of hydrogels containing GO depend both on the protein matrix and graphene oxide. Graphene oxide has two absorption bands in the UV (ultraviolet) region, with a tail extending into the visible [73]. The main absorption peak of GO, due to π→π * (from π-bonding orbitals to π *-antibonding orbitals) electronic transitions, is located at ~230 nm and occurs due to aromatic C=C bonds with a shoulder at ~300 nm, attributed to the n→π * (from non-bonding orbitals to π *-antibonding orbitals) electronic transition in the C=O functional groups from the surface of GO. The absorption spectra of graphene oxide depends on the number of graphene layers; in the case of multilayered GO, a broader UV-VIS absorption band can be observed compared with single-layered GO [74]. Proteins also have a strong absorption peak in UV-VIS at 280 nm [75] due to the presence of amino acids with aromatic rings [76]. 

**Figure 1:**
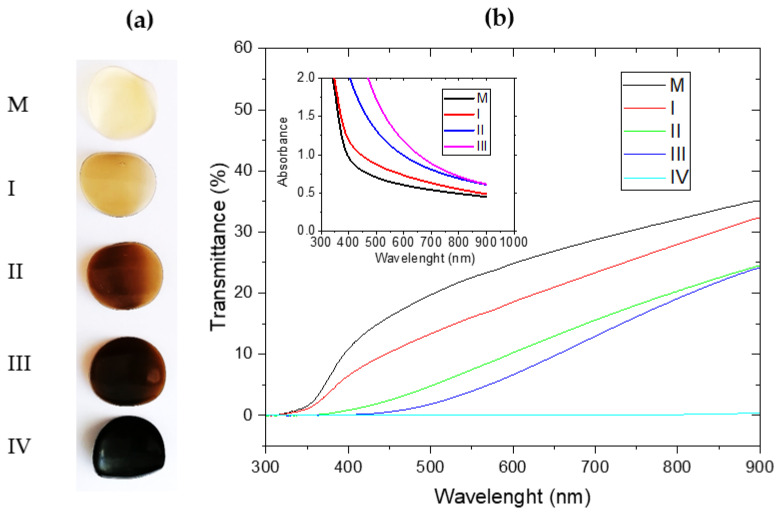
(**a**) Photo of hydrogel samples appearance from authors previous article [7], and (**b**) spectra of UV-VIS light transmittance of composite hydrogel samples: control sample (M), whey-based hydrogel reinforced with increasing GO concentration (I, II, III, and IV).

The appearance of hydrogel samples with and without GO, as shown in Figure 1a, was modified as the GO concentration increased from 0 to $33.33\times 10^{-3}$% and one could observe how the samples changed from transparent yellowish-white (M) to beige (I), caramel (II), brown (III), and finally black (IV), with a gradual loss of transparency to complete opacity for sample IV, the one with the maximum GO concentration. The UV-visible light transmission spectra of the hydrogel samples were measured, as shown in Figure 1b, to investigate their optical properties. The results were consistent with the above observations: (i) The control sample and sample I exhibited transmittance from wavelengths of ≈ 350 nm whilst sample II exhibited transmittance at ≈ 400 nm and sample III at ≈ 450 nm, indicating a poor visible light barrier. (ii) The wavelength range 350–400 nm, on which the control sample and sample I showed transmittance spectra, was due to the predominant gelatin [77] and whey [75,78] content, as observed in other research. (iii) Sample IV, being opaque, showed no transmission at all. The effect of GO on blocking UV and visible light transmittance has also been obtained in other hydrogel formulas such as chitosan/graphene oxide [79]. This feature may be useful for the packaging of light-sensitive food.

All hydrogel samples, with or without GO reinforcement, demonstrated good UV light barrier properties, deduced by the lack of transmittance in the UV range up to a wavelength of about 350 nm as a consequence of the high content of aromatic amino acids in the protein structure [80,81] and graphene. Therefore, of all five samples, the one with the highest concentration, $33.33\times 10^{-3}$%, conferred barrier properties to the hydrogel throughout the UV-VIS range. This hydrogel formulation may open new research perspectives in the field of light-sensitive food packaging. 

In the insert of Figure 1b, it is evident that the absorbance of the sample was highly dependent on the concentration of GO. In the case of sample IV, the absorbance was higher than 2 for all wavelengths; the spectrum of this sample is missing from the figure. The high content of GO randomly distributed in the protein matrix absorbed both UV, visible, and NIR light. At a high concentration of GO, the absorbance of the samples may also have been influenced by the structure of the matrix, which changed as the concentration of GO increased, as shown in Section 2.2.

### 2.2. X-ray Diffraction

**Figure 2:**
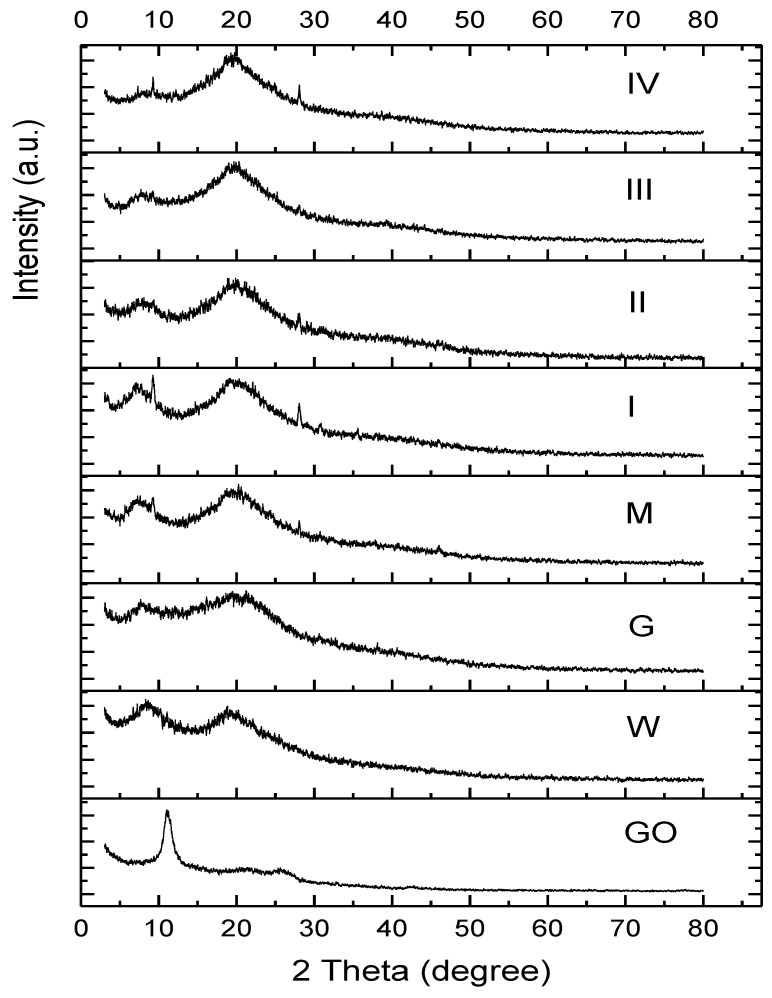
XRD spectra of starting compounds (whey (W), gelatin (Ge), and graphene oxide (GO)) and hydrogel samples: control sample (M) and samples with increasing concentration of GO (I, II, III, and IV).

The X-ray diffraction pattern for the starting compounds and hydrogel samples are shown in Figure 2. The X-ray diffraction spectra of GO had three broad diffraction peaks. The sharp peak at 2*θ* ≈ 11° corresponded with the crystallographic plane (001), which had an interplanar distance *d* ≈ 8 Å. Close to this value, at 2*θ* ≈ 11.8°, Soleimani et al. [8] identified a GO diffraction peak attributed to the graphite oxidation process. In correspondence with the crystallographic plane (002), a peak was observed at 2*θ* = 21.5° with an interplanar distance *d* ≈ 1.95 Å, and another peak at 2*θ* ≈ 25.9° with an interplanar distance d ≈ 1.71 Å. As this was the maximum of the second order, the distance between the layers was 3.42 Å.

The full width at half maximum (FWHM) of graphene oxide for the peak at 2*θ* ≈ 11° corrected by the instrumental width was *β* = 1.160. With the help of Scherrer’s relation (1), the crystallite size perpendicular to the (001) plane could be calculated. By transforming *β* into radians and performing the calculations, *D* = 69 Å was obtained.

(1) $$D=\frac{0.9\times \lambda }{\beta \times cos\theta }\;(Å)$$

The average number *n* of graphene layers could be calculated using Formula (2), where *d* was the distance between layers; in our case, 8 Å. Therefore, *n* = 8.6 layers.

(2) $$n=\frac{D}{d}$$

The crystallinity degree *CD* (%) of the hydrogels was calculated based on the surface of the diffraction peaks with the relation:

(3) $$CD=\frac{A_{c}}{A_{a}+A_{c}}\times 100\;(\%)$$

where *A_c_* is the surface area below the highest intensity peaks (crystalline structure) and *A_a_* is the surface area below the lowest peaks (amorphous).

Gelatin (Ge), whey (W), and the control sample (M) as well as samples with GO (I–IV) presented diffraction peaks (diffraction halos), whose approximate position (2*θ*, *d*) and corresponding area of each peak are presented in Table 1. The diffraction peak at 2*θ* ≈ 8° corresponded with the distance between each turn of the helical structure for gelatin [70] and whey; the area of this peak can be used as a measure of the relative helix content [71,72]. The peak at 2*θ* ≈ 20° was associated with the spacing between polypeptide chains [82]. It could be seen that the position of the two diffraction peaks from whey and gelatin did not differ too much. Other authors have found quite similar positions for the two peaks [82,83,84]. These results demonstrated that GO modified the structure of proteins in the composite hydrogels by decreasing the distances between turns and their corresponding areas with the passage from sample I to IV whilst increasing the distances between the polypeptide chains and their corresponding areas.

The full width at half maximum (FWHM) did not significantly change from one sample to another, and was approximately 2.6° for the first peak and 6° for the second peak. Using Scherrer’s relation, the length of the protein chain was estimated to be about 30 Å, and the transverse bundles of the associated polypeptide chains had an approximate average thickness of 13.2 Å. The fact that the area of the second diffraction peak increased when going from sample I to IV indicated that there was a process of association in the bundles of polypeptides.

In addition to the diffraction peaks described above, other diffraction lines of low intensity also appeared at angles 2*θ* ≈ 9.2°, 20°, and 28°, especially in samples M, I, and IV. In sample M, a peak at 2*θ* ≈ 46° was also observed. The diffraction peaks were due to the crystallization tendency of these proteins. Other authors have reported a tendency of the formation of new crystalline structures into the structure of hydrogels based on gelatin in the presence of a low concentration of an amorphous component (tannin) [85]. The appearance of small peaks in the case of the whey precursor was also observed at 2*θ* ≈ 10°, 20°, and 28° and the range of 35° to 40° by Aziz and Almasi in their work [86].

It was observed that the spectra of the GO-reinforced hydrogel based on whey and gelatin showed no peaks, referring to the presence of GO in their composition, probably due to its amorphous structure.

The degree of crystallinity of the hydrogels was calculated, based on the area of the diffraction peaks from Table 1. One could observe that the gelatin had a degree of crystallinity (18.47%) smaller than the crystallinity of whey proteins (34.43%). The hydration of proteins from the gelatin and whey during the formation of hydrogels led to the renaturation of helical structures in the resulting hydrogels, obtaining a crystallinity of 21.27% in the sample containing no GO. The degree of crystallinity of the GO containing hydrogels decreased with the increase in GO content from 20.73% for the sample containing $0{.66\;\times \;10}^{-3}$% GO to 12.72% (for the sample containing $3{.33\;\times \;10}^{-3}$% GO), to 9.83% (for the sample with $6{.67\;\times \;10}^{-3}$% GO), and to 7.14% (for the sample with $33{.33\;\times \;10}^{-3}$% GO). The decrease in the crystallinity of the hydrogel structures could be explained by the interactions between the GO through the functional groups from the surface of the graphene and proteins chains, which prevented (to a measure) the formation of ordered helical structures, characteristic for both gelatin and whey. More than that, the increase in the GO content resulted in a decrease in the distance between the turns of the helical structures. A similar situation has been observed in the case of Cu (II)-cross-linked hydrogels [14] when the increase in the Cu(II) content determined a decrease in the size of the helical structures. Although the interactions of Cu (II) ions with protein chains resulted in the formation of amorphous hydrogels with no helical structures at a high concentration of copper sulphate (0.7%), the interaction between carbon-containing compounds and protein chains was not as strong as ionic interactions involving Cu [14] of Ca [17,87] ions, as helical structures still formed in all samples of our GO-containing hydrogels (see Table 2). Similar results have been obtained for hydrogels containing whey and carbon nanotubes or nano-onions, whose diffraction patterns proved that secondary helical structures were formed in their hydrogels [88]. In conclusion, GO flakes influenced the crystallinity degree of the hydrogels and UV-VIS light barrier properties, although all samples maintained their crystalline phase.

### 2.3. FT-IR Spectroscopy

**Figure 3:**
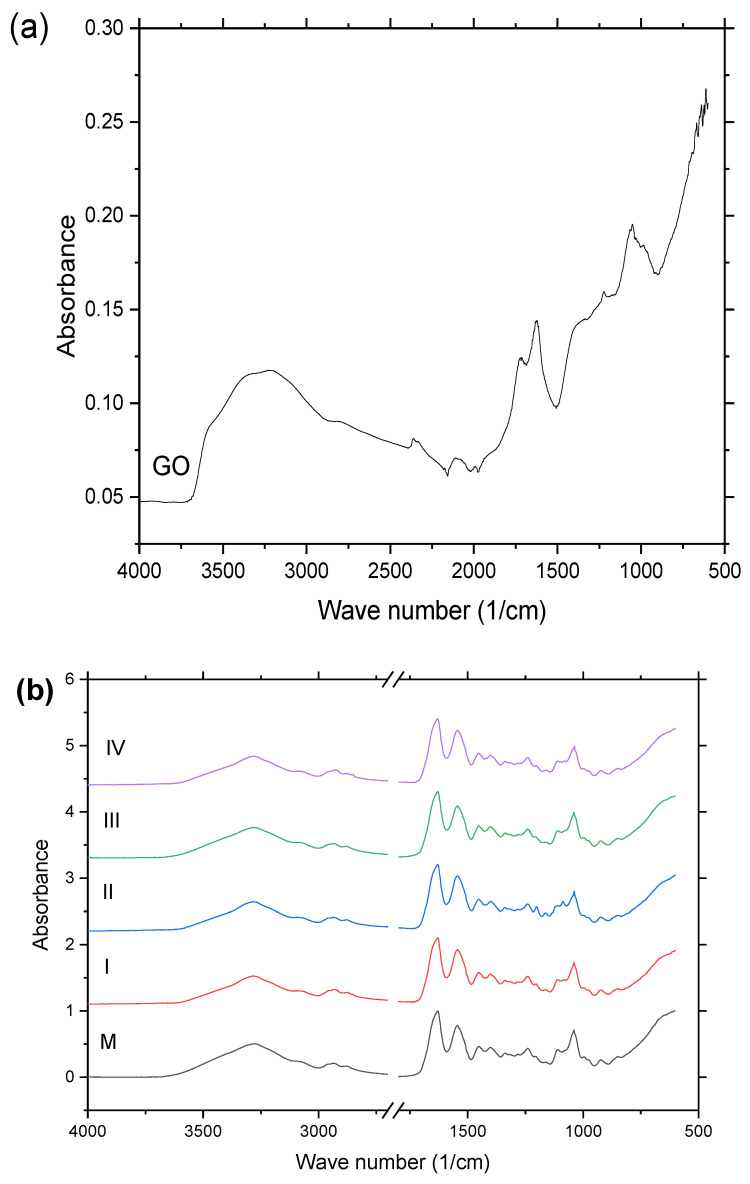
(**a**) IR spectra of graphene oxide (GO), (**b**) IR spectra of hydrogels with increasing GO concentration (I, II, III, and IV), and without GO (M).

To determine the changes in the structure of the gelatin and whey proteins as a result of their interaction with oxidized graphene, an FT-IR analysis was performed. Figure 3 shows the FT-IR spectra of the graphene oxide, control sample, and samples with an increasing GO concentration. Table 2 presents the main vibration bands of the precursors (whey and gelatin) and hydrogels, from which the data for gelatin were provided by previous authors’ work [14]. The GO samples showed distinct peaks of the absorption bands in the 1700 to 1500 cm^−1^ and 1100 to 1000 cm^−1^ regions, which could be assigned to polycyclic aromatic hydrocarbon bonds such as the stretching of C–H and C=O bonds, and 1 broad peak at around the 3280 cm^−1^ wavelength due to the O–H vibration. 

The band shifting of the N–H bond from the hydrogel spectra (Table 2) suggested that the graphene oxide interacted with the amino functional groups, forming new hydrogen bonds. The influence of the plasticizer (glycerin) was observed in the new absorption bands, related to C–OH from the glycerin molecule at the wavelengths ≈ 1038–1040 cm^−1^ for GO-reinforced hydrogels based on whey and gelatin.

One could conclude that, following the addition of GO into the hydrogels, no new chemical bonds were formed. The interaction of the protein chains with graphene oxide took place due to the formation of hydrogen bonds involving the functional groups from the surface of GO (C−OH, C−O−C, C=O, and O=C−OH). Although the new-formed bonds were not so strong, they influenced the protein renaturation process, leading, as was shown in Section 2.2, to less organized structures. However, as presented in a previous study [7], GO played the role of cross-linker in the obtained hydrogels.

The results were in good agreement with the results obtained from the X-ray diffraction measurements.

### 2.4. Morphology of GO and Hydrogel Samples with and without GO by TEM and SEM 

**Figure 4:**
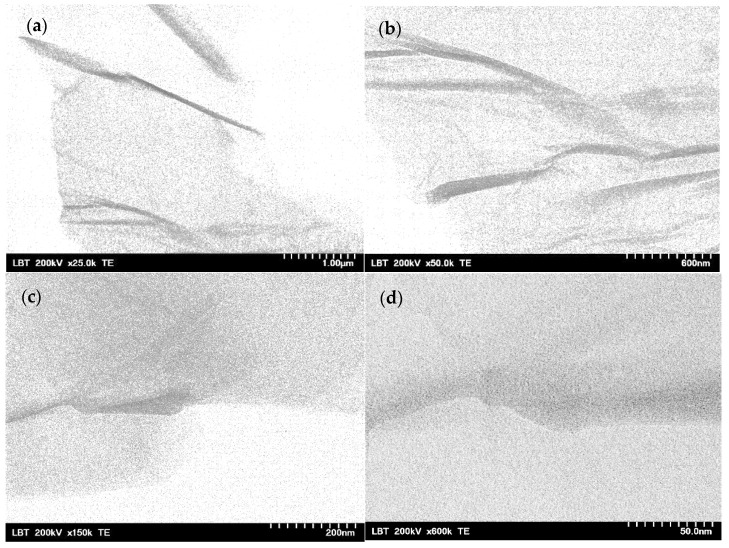
TEM images of GO suspension at various degrees of magnification: ×25.0 k (**a**), ×50.0 k (**b**), ×150 k (**c**) and ×600 k (**d**).

In Figure 4a,b, the distinct morphology of the GO sheets can be seen as a creased sheet aspect. The darker, somewhat linear-shaped regions suggest a folding or bending of the sheets, causing multilayered fragments of GO, whereas the lighter areas are more transparent due to a smaller number of layers of the analyzed sample [90]. This finding correlated with the diffraction analysis, which determined an average of 8 layers of the GO from its XRD pattern. The shades observed at higher magnifications in the TEM images of Figure 4c,d indicated the heterogeneous and rather disordered morphology of the GO sheets, reflecting the amorphous structure observed in the GO X-ray diffraction spectra in this study. A similar sheet shape morphology with various transparent regions was reported for graphite oxide [91] and graphene oxide with different degrees of oxidation [90].

**Figure 5:**
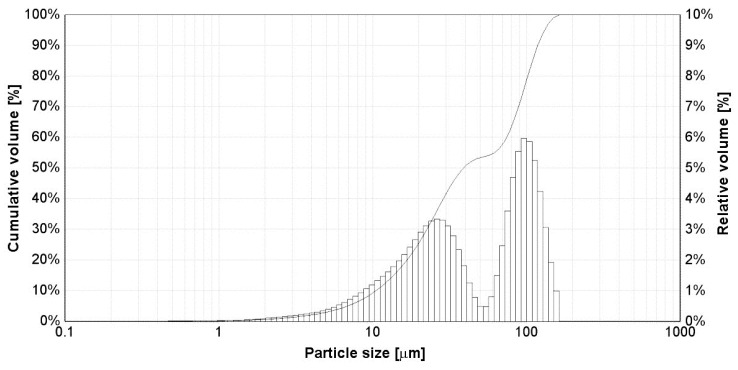
The size distribution of graphene oxide powder particles obtained by laser analysis.

The size distribution of the graphene oxide powder particles obtained by a laser analysis is presented in Figure 5 in the form of a histogram with the weight of the granulometric classes as well as a cumulative transition curve. According to the results, the size of the powder particles was between 0.3 and 167 µm and the particle size distribution was bimodal, with the maxima centered on the 20–30 µm and 90–100 µm classes. The values of the D10, D50, and D90 parameters were 10.51, 39.11, and 118.65 μm, respectively. Graphene oxide tends to agglomerate in an aqueous dispersion medium even under the action of ultrasound [92] due to the interaction between the functional groups from the surface of graphene due to electrostatic forces [93]. Other authors have obtained a modal size of 90.83 µm for an electrochemically synthesized multilayer [92].

**Figure 6:**
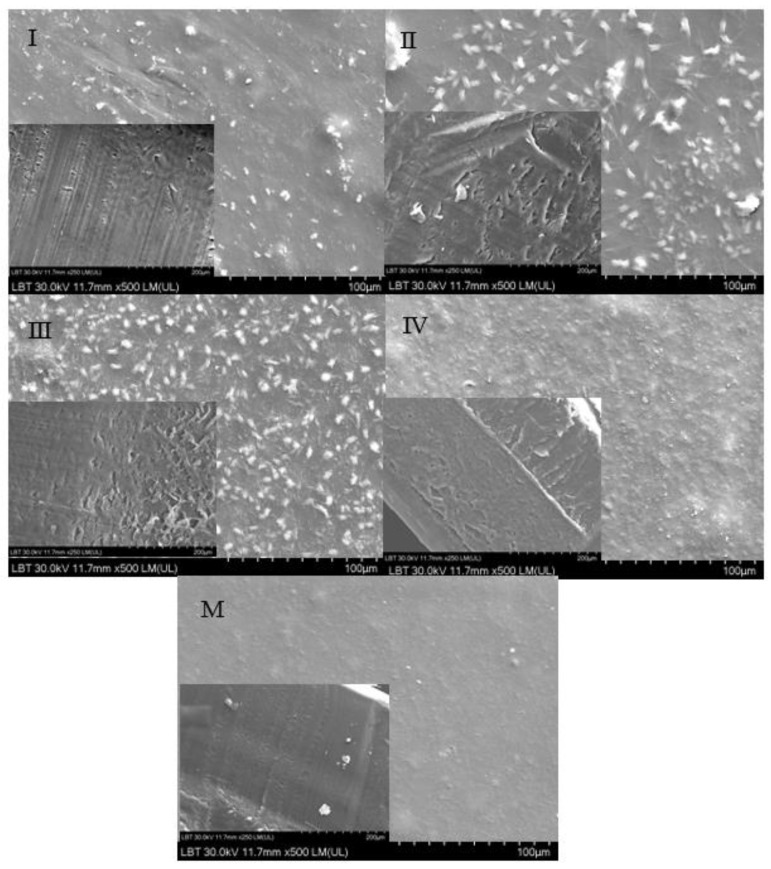
SEM images of the surface (big picture) and the cross-section (inside smaller picture) of hydrogel samples based on whey and gelatin without GO (M) and with increasing GO concentrations (I, II, III, and IV).

The morphology of the hydrogel surfaces can be seen in Figure 6 in the larger SEM images, and the cross-section of each hydrogel sample can be visualized in the smaller images superimposed over the larger ones. The white dots, representative of insoluble gelatin particles [94], were more pronounced in the hydrogels with GO than in the reference sample (M). Their uniform distribution in hydrogel samples III and IV was an indicator of the homogeneous dispersion of GO in the hydrogel’s matrix, preventing the gelatin particles from aggregating, as particularly observed in sample II. The lack of cracks on the surface of the hydrogels and their continuous appearance indicated a good embedding of GO in the polymer matrix [94] due to its hydrophilic nature, which allowed it to have strong bonds through oxygen-rich functional groups with the functional groups of the whey and gelatin proteins. Therefore, a significant cross-linking effect was generated, which also led to the increased stiffness of sample IV. As can be seen in the small images placed in the lower left corners over the larger images in Figure 6, the control sample (M) showed no distinct porosity compared with the GO samples where the number and the size of pores increased with an increasing GO concentration. A similar pore pattern could be seen in the SEM images of a hydrogel based on gelatin, sodium alginate, and GO [10]. It could be concluded that the cross-linking of the hydrogel in this study with the maximum GO content led to an improvement in its resistance in an aqueous medium as well as its antibacterial properties, as determined in the previous study of the authors [7]. By correlating these results with the optical barrier properties resulting from the UV-VIS spectroscopic analysis, a material for packaging light-sensitive food could be modeled.

### 2.5. Swelling Assessments by Electric Conductivity 

**Figure 7:**
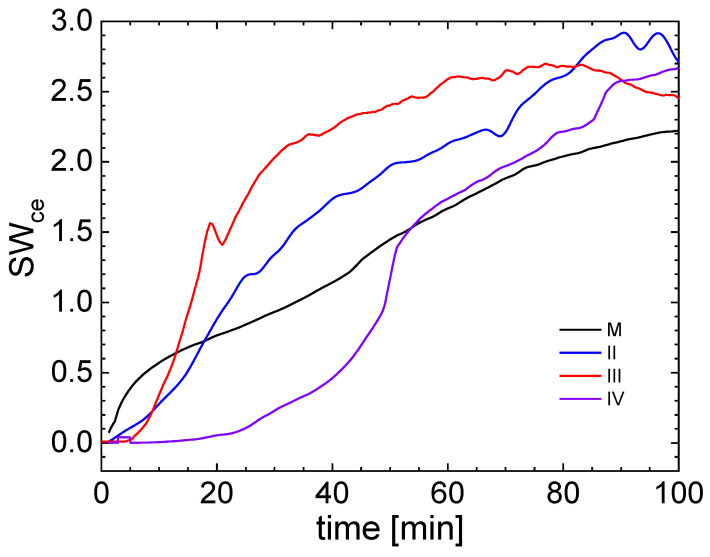
Rescaled swelling ratio evaluated from electric conductivity measurement in the initial time regime for the control sample M and samples with an increased quantity of GO (II, III, and IV).

The swelling curves obtained from rescaling the electric conductivity variation in the immersed hydrogel samples with or without graphene oxide are presented in Figure 7. By monitoring the electric conductivity behavior of the hydrated hydrogel, it was observed that, at least in the initial time regime, the value of the electric conductivity varied with the variation in the amount of swelled distilled water. For the dry samples, the values of electric conductivity were close to zero; the samples were a relatively good isolator. Once this sample came in contact with water, the electric conductivity of the hydrogel started to increase. One could also assume that the air humidity played a small role in the initial (not immersed) value of the electric conductivity measured for the dry samples. The behavior of the control sample (M) and hydrogel samples with the increased amounts of graphene oxide in II, III, and IV were as expected, but the behavior of the hydrogel sample with the smallest amount of graphene oxide (sample I) was different; it will be discussed later.

Immediately after immersion, as expected, the control hydrogel sample (M) started to absorb the distilled water, as can be observed from the abrupt increase in the black curve in Figure 7. Within 10 min, the amount of absorbed water was approximately 60% of the hydrogel mass. In time (in the initial time regime, up to 100 min), the ratio of the swelling characteristic to the hydrogel of M became smaller. The hydrogel sample II in the first 8 min of swelling presented the second velocity of swelling (after sample M). In the first 5 min, the hydrogel of samples III and IV (with the highest amount of GO and the largest rigidity) absorbed only a small amount of distilled water. Sample III then started to absorb the largest amount of distilled water, as can be observed from the variation in the red curve in Figure 7. At approximately 9 min after immersion, the swelling ratio value measured for sample III was the same as the swelling ratio measured for sample II, and at approximately 13 min from immersion, the swelling value of sample III matched the value measured for the control (M) hydrogel. One could then consider that the swelling rate characteristic of hydrogel III was the largest. The swelling value for hydrogel III was the largest up to approximately minute 82, when it was matched by the value measured for sample II. Moreover, one could observe that the characteristic swelling value measured for sample II matched the measured value for the control sample at approximately minute 19. Approximately 20 min after immersion, hydrogel sample IV, with the largest amount of GO, absorbed just a small amount of distilled water; an increased swelling ratio was later observed for this hydrogel in the time interval from minute 40 and 50. This was probably due to the increased polymer network rigidity induced by the highest amount of GO. The swelling value measured for hydrogel IV matched the measured value for the control hydrogel (with no GO) approximately 54 min after immersion, and one could observe that the match with the swelling value measured for sample III was approximately at minute 90 and, with the measured value for sample II, at minute 100.

As mentioned before, the swelling amount of these samples, measured by electric conductivity, at the time moment of 100 min from immersion was the same as the swelling amount measured by the classical weighing method (presented and characterized in previous works [7,14]). Briefly, for a long immersion time, one could observe that the final swelling amount was measured for the hydrogel sample with no graphene. With the increase in GO content, the final swelling amount decreased with the increase in the graphene oxide content. The accuracy of the swelling measurement via electric conductivity decreased with time, mainly due to the fact that, by swelling, the integrity of the polymer network was affected because the hydrogel started to lose mass. In the medium- and long-time regimes, one could then observe a decay in the electric conductivity, which no longer reflected the amount of absorbed water. 

**Figure 8:**
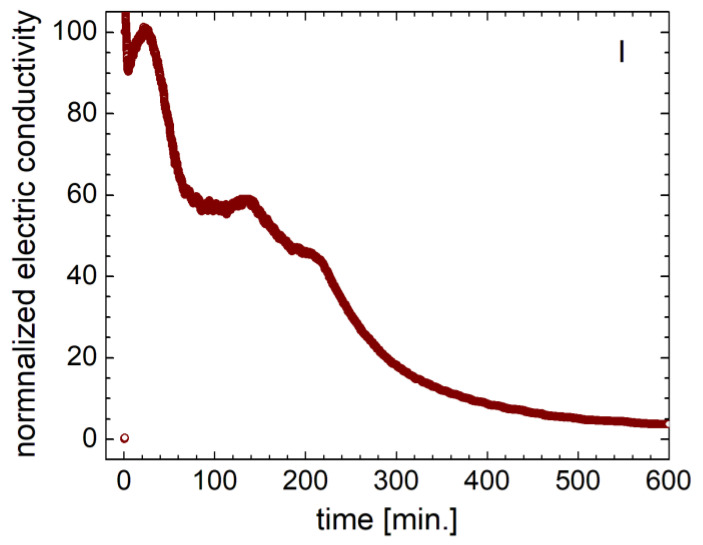
Normalized electric conductivity measurement for sample I with the smallest content of GO.

A different electric conductivity behavior was recorded for hydrogel sample I, characterized by a smaller amount of GO, and is presented in Figure 8. The dry sample was characterized by negligible electric conductivity. Immediately (1–2 s) after the hydrogel touched the distilled water, the electric conductivity dramatically increased. This behavior clearly could not be attributed to absorbed water. The measurement was repeated several times, but the same behavior was observed. A dramatic increase occurred in electric conductivity in an extremely short time (compared with the dwell time, the time between the two measurements), and then the electric conductivity started to immediately decay into a multiexponential decay interrupted by several peaks or shoulders. This was an indication of the structural modifications at the level of the hydrogel polymer network.

## 3. Conclusions

The study proposed a completely new composite hydrogel formulation with whey and gelatin as protein precursors cross-linked with graphene oxide in the range of $0.66\times 10^{-3}\;\mathrm{and}\;33.33\times 10^{-3}$ concentrations. Increased stiffness and opacity and a color shift from yellowish-white to black with the GO concentration were easily observed, even on visual and tactile examinations. Color shading associated with a lower transparency corresponding with the GO content was also observed in the UV-VIS spectra, whose absorption band intensity decreased with an increasing GO content. These results were consistent with the cross-linking effect of GO evident in the XRD spectra, which showed a decrease in the distances between the polymer chains in the hydrogel matrix structure produced by the cross-linker, and the FT-IR spectra, where shifts in the absorption bands of the functional groups were revealed in the structure of the whey and gelatin proteins due to the new hydrogen and covalent bonds created with GO. The micrometric size of GO corroborated the multilayer structure of this precursor, identified by both X-ray diffraction and TEM analyses. At these dimensions, the GO interacted with the hydrogel matrix through hydrogen bonds, as revealed by the FT-IR results, occupying the free spaces between the polymer chains. The effect of this interaction manifested itself both in the stiffening of the hydrogels and in the decreased transmittance in the UV-VIS range.

The electric conductivity method was successfully used for the assessment of the initial time regime of the distilled water-swelling degree for a few hydrogel samples with and without graphene, with one exception represented by the hydrogel sample characterized by the smallest amount of graphene. The experimental set-up for the electric conductivity allowed sampling with a time interval of approximately one second with no intervention on samples, as in the case of classical measurements by weighing (which also implies a procedure of sample swapping). This direct and fast monitoring is extremely important in the case of fast processes, as in the case of water swelling by hydrogels. Therefore, it was shown that the water-swelling process, monitored via electric conductivity, was not monotone and multiple mechanisms (characterized by different swelling velocities) were observed. The dry hydrogel sample with a small amount of graphene was extremely sensitive to water, as observed from the electric conductivity measurements, presenting a real potential to be part of a specialized sensor.

These findings may provide an opportunity for the development of a whey–gelatin-based composite hydrogel reinforced with graphene oxide and blended with antibacterial compounds in order to obtain swelling and antibacterial activity for an eco-friendly absorbent material for high moisture content food packaging.

## 4. Materials and Methods

### 4.1. Materials

Four hydrogel samples based on whey–gelatin with increasing concentrations of GO and a control sample (M) were prepared, as described in the authors’ previous article [7]. Instant whey protein isolate (WPI) active, produced and distributed by Sly Nutritia SRL (Buzău, Romania), and a gelatin reagent from AMRESCO, LLC (Fountain Parkway Solon, OH, USA) were used for the polymeric matrix. The plasticizer used was glycerol, produced by Sigma-Aldrich (Taufkirchen, Germany), and the reinforcing agent, graphene oxide, was obtained by Hummer’s method from graphite by the Polymeric Composite Laboratory of the Institute of Chemistry Raluca Ripan (Cluj-Napoca, Romania), as described in [95]. 

One of the objectives of this hydrogel formulation is to have an optimal swelling capacity and a slightly flexible consistency. Concentrations of GO were studied over the range 0–$50\times 10^{-3}$%, and the results, which were consistent with the objectives of this study, led to the four concentrations presented in this article and in the authors’ previous work [7]. The samples with concentrations exceeding 33.33 $\times 10^{-3}$% GO had the lowest swelling degree, even though the antibacterial properties were superior to the other samples with a lower GO concentration. However, natural antimicrobial agents can be blended in the hydrogel formula, as shown by the results of numerous food packaging research studies.

The percentage ratio of GO in the composite hydrogel samples is shown in Table 3.

### 4.2. UV-VIS Spectroscopy

UV-VIS spectra of the hydrogels were measured on transmission mode using a double-beam UV-VIS spectrometer (Lambda 35) produced by Perkin-Elmer (Waltham, MA, USA) in the 900–350 nm wavelength range. For the UV-VIS spectra measurements, solid sample holders provided with homemade additional supports for the hydrogels were used. The blank measurement was made without samples [15]. 

### 4.3. X-ray Diffraction

The X-ray powder diffraction patterns were scanned using a Bruker D8 Advance diffractometer in Bragg–Brentano reflection geometry with a Cu tube powered at 40 kV and 40 mA. A Ge (1 1 1) monochromator was mounted in the incident beam in order to obtain only CuKα1 monochromatic radiation, with λ = 1.54056 Å, and the diffracted radiation was recorded with a LYNXEYE position detector. The scan was performed in the 3–80° angular 2*θ* range, with a scan rate of 0.02 degrees/s [96].

### 4.4. FT-IR Spectroscopy

For the recording of the FT-IR spectra of hydrogels, the samples were dried to a constant weight at room temperature. A Spectrum BX FT-IR spectrometer (Perkin Elmer, Sunnyvale, CA, USA), provided with an attenuated total reflectance (ATR PIKE MIRacleTM, Madison, WI, USA) with a diamond crystal plate, was the used for the absorbance measurements, with a resolution of 2 cm^−1^ in the range of 4000–500 cm^−1^ [97]. The position of each sample on the surface of the ATR windows was changed until a clear, well-defined FT-IR spectrum was obtained for each sample. All FT-IR spectra were normalized for amide I band.

### 4.5. TEM and SEM 

The study of the microstructure and morphology [10,94] of the control sample and the ones with increasing GO concentrations was conducted with the help of an Inspect-S microscope (FEI Company, Hillsboro, OR, SUA). Samples were sputter-coated with 10 nm Au (Agar Automated Sputter Coater, Essex, UK) and images recorded at 30 kV with a HRSTEM Hitachi SU8230 (Hitachi, Japan). Separately, GO was suspended in ethanol and sonicated prior to the deposition of 7 nm Ultra-Thin (UL) onto a carbon-coated 400 mesh Ni grid. Images were recorded on a Hitachi HD-2700 UHR CFEG STEM (Hitachi, Japan) at 200 kV. The particle size distribution of the graphene oxide powder was determined using an Analysette 22 NanoTec laser analyzer produced by the Fritsch company (Idar-Oberstein, Germany) using water as a dispersion medium [98]. To ensure a stable suspension during the laser analysis, the graphene oxide powder was dispersed in water at a concentration of 4% (weight%) and ultrasonicated for 8 h.

### 4.6. Swelling Assessments by Electric Conductivity

For the evaluation of the swelling ratio via the measurement of the variation of the electric conductivity in the initial time regime, a homemade device was constructed. This was based on the data acquisition using an Arduino UNO microcontroller and soil humidity sensors, for which the regular electrodes were replaced with clamps. The signal was first passed to an LM393 comparator placed on a PC board, on which the potentiometer could be used for a sensitivity adjustment. The signal received by the Arduino UNO was analog, between 0 and 5 V, and converted to digital numbers by a 10 bite ADC (analog to digital converter). Additionally, a DHT22 digital humidity and temperature sensor was placed in the immediate vicinity of the electrodes for the monitoring of the environmental properties. The electrodes were placed on a stand with arms. A Berzelius beaker with distilled water was placed immediately below them. The hydrogel samples were cut in a rectangular shape with dimensions of 40 × 10 mm and a thickness of 1.2 mm, with an error of less than 5%. The clamps clamped 5 mm of each sample from each end; these were then brought together to about 10 mm so that the sample was U-shaped and vertically oriented. The entire ensemble could be vertically displaced so the hydrogel samples could be placed in distilled water without the electrodes being effectively introduced into the distilled water. The electric conductivity, electric resistance, air humidity, and temperature were measured and transmitted via the serial port to the PC with a frequency of approximately one second. A dedicated program was written in Processing, which graphically represented the evolution of the electric conductivity in time and wrote all measured values into files. For the resistance measurement, the electric voltage was calibrated using a series of standard resistances, but, in this case, this information was not used. The samples were weighed prior to and after the electric conductivity measurements, but because a maximum of the electric conductivity measurement was reached for each sample at different times, the continuous measurement of the swelling ratio was used instead [7,14] at 100 min. 

## Figures and Tables

**Table 1:** Area of peaks and distance between turns in helical structure and polypeptide chains corresponding with 2*θ* angles.

Sample	2*θ* (Degree)	Distance between Turns (Å)	Area (a.u.)	2*θ* (Degree)	Distance between Chains (Å)	Area (a.u.)	Crystallinity (%)
Ge	7.7	11.5	123	20.5	4.33	543	18.47
W	8.4	10.5	167	19	4.66	318	34.43
M	7.7	11.5	191	20.2	4.39	707	21.27
I	7.3	12.1	200	19.4	4.57	765	20.73
II	7.9	11.2	87	19.9	4.46	597	12.72
III	7.9	11.2	91	19.6	4.52	835	9.83
IV	8.8	10.0	67	19.6	4.52	872	7.14

**Table 2:** The main vibration bands of GO-reinforced hydrogels based on whey and gelatin and their components.

Gelatin	Whey	M	I	II	III	IV	Attributions
[cm^−1^]							
3272.82	3272.4	3281	3284	3285	3285	3285	Amide A; tensile vibrations NH [14,17,71]; hydrogen bonds [14,15]
1630.62	1631.72	1630	1630	1630	1630	1630	Amide I; C=O and NH vibrations [14,71,86]; H bonds coupled with COO^−^ [15,87]
1527.47	1515.90	1544	1544	1544	1543	1543	Amide II; CN vibrations/stretching; NH bending [14,15,89]
1080.02	1076.17	1079	1083	1087	1079	1081	Amide III; CN and NH vibrations [14,15,89]

**Table 3:** Percentage composition of graphene oxide in whey–gelatin-based hydrogels.

	M	I	II	III	IV
GO (%)	0	$0.66\times 10^{-3}$	$3.33\times 10^{-3}$	$6.67\times 10^{-3}$	$33.33\times 10^{-3}$

## Data Availability

Not applicable.
